# Recognition of the Duration and Prediction of Insect Prevalence of Stored Rough Rice Infested by the Red Flour Beetle (*Tribolium castaneum* Herbst) Using an Electronic Nose

**DOI:** 10.3390/s17040688

**Published:** 2017-04-14

**Authors:** Sai Xu, Zhiyan Zhou, Keliang Li, Sierra Mari Jamir, Xiwen Luo

**Affiliations:** 1 South China Agricultural University/Engineering Research Center for Agricultural Aviation Application (ERCAAA), Guangzhou 510642, China; xusai1991@sina.cn (S.X.); 18825179816@163.com (K.L.); xwluo@scau.edu.cn (X.L.); 2 Collaborative Innovation Center for Grain and Oil Crops in South China, Changsha 410128, China; 3 Department of Food Science, Cornell University, Stocking Hall, Ithaca, NY 14853, USA; sierrajamir@gmail.com

**Keywords:** rough rice, storage, electronic nose, red flour beetle, duration, insect prevalence

## Abstract

The purpose of this research is to explore the feasibility of applying an electronic nose for the intelligent monitoring of injurious insects in a stored grain environment. In this study, we employed an electronic nose to sample rough rice that contained three degrees of red flour beetle (*Tribolium castaneum* Herbst) infestation for different durations—light degree (LD), middle degree (MD), and heavy degree (HD)—and manually investigated the insect situation at the same time. Manual insect situation investigation shows that, in all three rice treatments, the insect amounts gradually decreased after infestation. When the insect population of stored rough rice was under 13 insects per 60 g of rough rice, the natural speed of decrease of the insect population became very slow and reached the best artificial insect killing period. Linear discriminant analysis (LDA) provided good performance for MD and HD insect harm duration identification, but performed poorly for LD insect harm duration identification. Both k-means clustering analysis (K-means) and fuzzy c-means analysis (FCM) effectively identified the insect harm duration for stored rough rice. The results from the back-propagation artificial neural network (BPNN) insect prevalence prediction for the three degrees of rough rice infestation demonstrated that the electronic nose could effectively predict insect prevalence in stored grain (fitting coefficients were larger than 0.89). The predictive ability was best for LD, second best for MD, and least accurate for HD. This experiment demonstrates the feasibility of electronic noses for detecting both the duration and prevalence of an insect infestation in stored grain and provides a reference for the intelligent monitoring of an insect infestation in stored grains.

## 1. Introduction

Rice is the most important crop in China. Approximately 65% of Chinese people live on rice. China is also the largest rice-producing country in the world, amounting to approximately 30% of the world’s total production [1]. Pest insects are one of the main factors that cause grain loss. Researchers have reported that [2] 5% of the total grain in the world is lost due to infestation by insects every year. If manpower, material resources, and technology cannot meet the needs of grain protection, losses can reach 20%–30% of total grain. Annual losses of grain depots in China were approximately 0.2% of total grain production. Pest insects must be accurately detected to purposely administer prophylaxis and treatment. Thus, real-time detection of insects for stored grain is an imminent problem that has not yet been solved. Today, there are several insect-detection methods, such as manual work detection [3], acoustical signal detection [4], image recognition [5], and near-infrared spectroscopy detection [6]. The manual detection work method requires large amounts of time and labor, and this method does not meet the requirements of intelligent detection. The acoustical signal detection method is often disturbed by sensor and environmental noise, among others. Further, acoustic signals of insects are often very weak and are easily drowned out by other noise. Finally, because insects are covered by stored grain, the image recognition and near-infrared spectroscopy detection methods are not well-suited for insect detection in granary environments. Thus, it is important to find an effective means for the intelligent detection of insects in stored grains. 

As a bionic olfactory system, the electronic nose is a comprehensive system that has several gas sensors to acquire the information “fingerprint” of the target’s volatile compounds [7]. Electronic noses are easy and quick to operate and are not influenced by the cover of stored grain when detecting insects. Compared with other detection methods, such as manual detection, acoustical signature detection, image identification, and spectrum detection, electronic noses can overcome all of their collective disadvantages and are more suitable for insect identification in storage environments. Electronic nose systems have already been applied in some fields, such as medical health [8], environmental monitoring [9], and food quality detection [10].

Several years of research have detected a total of 125 components in stored grain when infested by red flour beetles and grain borers, among others [11]. This research report provides a theoretical foundation for insect identification from the change of volatile compounds in stored grain. At present, electronic noses have already been successfully used for insect detection in stored grain. In 1993, Stetter et al. used an electronic nose to classify wheat samples by quality grade. They classified wheat as good, fermented, and damaged by pests with a success rate of 83% [12]. In 1999, Ridgway et al. demonstrated that electronic noses are useful for pest identification and discriminated between wheat samples with no mites and wheat samples with 70 added mites with a classification accuracy of over 83% [13]. In 2005, Jiang et al. adopted an electronic nose to detect standard air and volatile compounds of stored food with live and dead pests. Their results demonstrate that using an electronic nose to identify pests in stored food is feasible and that such a system can accurately detect pest status [14]. In 2007, Zhang et al. used an electronic nose to evaluate and classify five different storage ages of wheat with 15 degrees of insect damage. Their results indicated that the electronic nose could successfully discriminate among wheat samples of different ages and with different degrees of insect damage [15]. However, insect durations and prevalence monitoring of stored grains based on time series has not yet been reported.

Thus, rough rice samples infested by red flour beetles were monitored based on time series in this experiment. Sample information of rough rice at different times was acquired using the electronic nose and manual work detection methods. Linear discriminant analysis (LDA), k-means clustering analysis (K-means), fuzzy c-mean clustering (FCM), and back-propagation artificial neural networks (BPNNs) were used for pattern recognition. This study explored the feasibility of using an electronic nose to predict the duration and prevalence of insect infestation in stored grain and provides a reference for the intelligent monitoring of stored grain.

## 2. Materials and Methods

### 2.1. Experimental Materials

The experimental rough rice was of the “Meixiangzhan” variety and was harvested at a Baiyun test field, Zhongluotan, Guangdong province, China, in December 2014. After harvesting, natural drying under sunlight was applied until its water content stabilized at between 12% and 14% (the best water content for rice storage). Then, unbroken and undamaged rough rice samples were selected for the test. Each experimental sample contained 60 g of rough rice and was placed in a 200 mL glass beaker. All of the beakers were cleaned using an ultrasonic cleaner and air-dried in a room with no abnormal smells before adding rice. There was 1 control (no-treatment, NT) and 3 experimental treatments: light degree damage treatment (LD), middle degree damage treatment (MD), and heavy degree damage treatment (HD). Each treatment consisted of five replications. LD samples manually received 10 red flour beetles, each MD sample received 50 red flour beetles, and each HD sample received 100 red flour beetles. Each sample was placed in a plastic box (length × width × height = 23 × 17 × 14.5 cm). During storage, each sample was sealed by a gauze element, and the cover of the plastic box was closed. We began the first test 2 days before infestation (−2 d), and then 2 days, 7 days, 13 days, 22 days, 28 days, and 36 days after infestation (2 d, 7 d, 13 d, 22 d, 28 d, and 36 d). Both an electronic nose and manual work detection were employed.

### 2.2. Insect Situation Investigation

The manual work detection method was used in this experiment to investigate insect prevalence. To conduct the investigation, each rice sample was placed onto 2 sheets of white paper and the number of live insects was counted directly with the naked eye and with a magnifying glass. Afterwards, rice samples were sequentially replaced to their respective beakers for storage.

### 2.3. Electronic Nose Sampling

An electronic nose (PEN3 AIRSENSE, Inc., Schwerin, Germany) was used for sampling in this experiment. The electronic nose is mainly composed of a sensor array, a sampling and cleaning channel, a data processing system, and so on. The sensor array includes 10 metal oxide gas sensors. Each sensor is sensitive to different volatile compounds, which allows the electronic nose to detect complex smells. The detected compounds of the 10 sensors were as follows [16]: W1C (aromatic), W5S (broadrange), W3C (aromatic), W6S (hydrogen), W5C (aromatic-aliphatics), W1S (broad-methane), W1W (sulfur-organic), W2S (broad-alcohol), W2W (sulfur-chlorinate), and W3S (methane-aliphatics).

Before sampling, the gauze elements were removed from the beakers and the beakers were then sealed using double-deck plastic films for 1 h. The sampling parameter settings were as follows: the sampling interval was 1 s; flush time was 70 s; zero point trim time was 10 s; measurement time was 60 s; presampling time was 5 s; and injection flow was 300 mL/min.

This experiment acquired 140 electronic nose data (4 degrees of damage × 5 samples for each degree × 7 durations for each sample = 140) for different degrees of infested rough rice sampling. The data was obtained at 55 s for each sample was reported as the feature value.

### 2.4. Data Pre-Processing

Electronic noses have inevitably been reported as being influenced by drift noise when detecting based on time series [17]. This drift noise is usually caused by changes in environmental temperature and humidity, or by changes of sample itself. Thus, this experiment referenced research results of Yin et al. [18] to use the reference signal removal method for data pre-processing. Specifically, we used the electronic nose sampling data for LD, MD, and HD minus the average values of the 5 NT electronic nose sampling data at the same times, which effectively removed the drift noise signal of the same time but kept the useful signal only caused by insect infection. 

### 2.5. Data Processing Method

Linear discriminant analysis (LDA) is also a linear pattern recognition method that uses dimensionality reduction. LDA, however, focuses on the distributions and distances within each treatment. It can collect information from whole sensors and delineate each treatment using a particular vectorization transformation, which results in the samples within a treatment being condensed and distant samples being sorted in different treatments [19].

K-means clustering analysis (K-means) is a clustering method based on partitioning. It attempts to find the K best clustering centers via iteration, allotting all sample data points to K clustering centers and minimizing the sum of the square errors of clustering at the same time [20]. 

Fuzzy c-mean clustering (FCM) is an unsupervised recognition method of clustering that allows one data point to belong to two or more clusters. It is based on the minimization of the objective function Jm. m is the weighted index of Jm and is a real number greater than 1. The value of m decides the type of objective function. Thus, finding a suitable m and Jm values is important for accurate FCM classification [21].

Back-propagation artificial neural networks (BPNNs) are one of the most commonly used neural networks and include input, hidden, and output layers. In the process of training BPNNs for analysis, the weights and threshold values of each layer are constantly revised. BPNNs adjust the weights and threshold values repeatedly based on the differences between the expected outputs and actual outputs. Thus, a BPNN is a neural network that spreads information in the forward direction and returns the difference in the reverse direction. This training lasts until the difference between the expected outputs and actual outputs is reduced to a preset range or until the scheduled training times are achieved [22].

## 3. Results

### 3.1. Change of the Insect Number at Different Degrees and Durations of Infestation

Average numbers (rounded to whole numbers) of live insects for the five replications were used to represent the live insect number of each infestation degree at a specific time. The insect number changes of in the different infestation degrees of rough rice at different times are shown in Table 1. During injury, live red flour beetle numbers in all of the LD, MD, and HD samples gradually decreased. However, the speed of the decrease in LD was slow after injury and tended to stabilize after 28 d. The decreased speed in LD was rapid from 0 to 13 d after injury, but slowed after 13 d. The decreased speed in HD was rapid from 0 to 22 d after injury, but slowed down after 22 d. According to trends of insect variation in the different infestation treatments, we can infer that insect numbers naturally decline slowly when populations in storage grain are under 13 individuals/60 g, which is the necessary period for manual insect killing.

### 3.2. LDA for Duration Recognition in Different Infestation Degrees in Rough Rice

**Figure 1:**
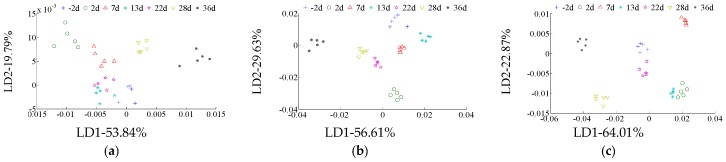
Linear discriminant analysis (LDA) for duration recognition of different infestation degrees in rough rice. (**a**) Duration recognition of light degree damage treatment (LD); (**b**) duration recognition of middle degree damage treatment (MD); (**c**) duration recognition of heavy degree damage treatment (HD).

LDA results for duration (−2 d, 2 d, 7 d, 13 d, 22 d, 28 d, and 36 d) recognition of different infestation degrees in rough rice are shown in Figure 1. The LDA results for infestation duration recognition of LD are shown in Figure 1a. The first linear discriminant factor’s contribution (LD1) is 53.84%, and the second linear discriminant factor’s contribution (LD2) is 19.79%, for a total contribution of LD1 and LD2 of 99.87%. All of the durations of infestation in rough rice did not overlap with others and could be classified. However, 13 d was close to 22 d and could be confused in practical classification and recognition. The LDA results for the infestation duration recognition of MD are shown in Figure 1b. LD1 contributed 56.61%, while LD2 contributed 29.63%, for a total contribution of LD1 and LD2 of 86.24%. All of the infested durations did not overlap and could be classified effectively. The LDA results for the infestation duration recognition of HD are shown in Figure 1c. LD1 contributed 64.01%, and LD2 contributed 22.87%, for a total contribution of LD1 and LD2 of 86.88%. All of the infested durations did not overlap with others and could be classified. Thus, the infested duration LDA classification effect is good in MD and HD, but poor in LD. This may be because the volatiles of LD (less infected) was lighter and changed slower than that of MD and HD.

### 3.3. Clustering Analysis for Duration Recognition of Different Infestation Degrees of Rough Rice

To further explore the duration recognition accuracy of different infestation degrees of rough rice, clustering analysis was applied in this research. So far, there are four commonly used clustering analysis methods, namely, K-means, hierarchical clustering, SOM clustering, and FCM. However, research has shown that K-means and FCM often have better classification abilities than the other two methods [23]. Thus, this experiment used K-means and FCM to further classify the duration of different infestation degrees of rough rice. In addition, research shows that extracting the linear discriminant factor matrices of the LDA results as feature values to replace the original ones can usually further reduce redundant information and yield a better classification effect [24]. Thus, this experiment used the linear discriminant factor matrix of the LDA results as feature values for further research to explore the feasibility of using an electronic nose for the duration recognition of different infestation degrees of rough rice. 

The classification results of K-means for the duration of different infestation degrees of rough rice are shown in Table 2. K-means for infestation duration recognition of LD, MD, and HD are 88.57%, 91.42%, and 91.42%, respectively.

When FCM was used for classification, weighted m values (weighted index of the objective function J_m_) had significant impacts on the classification results. After repeated analyses, the m values and classification results are shown in Table 3. FCM classification accuracies for the duration of LD, MD, and HD were 94.29%, 100%, and 100%, respectively. The classification abilities of FCM were better than the K-means for duration recognition of different infestation degrees in rough rice.

### 3.4. BPNN for the Prediction of The Live Insect Amount in Infested Rough Rice

We used BPNN to explore the feasibility of stored rough rice’s insect prevalence predictions based on an electronic nose. There are three infestation degrees included in the analysis, namely, LD, MD, and HD. Each treatment included five samples. Thus, there were 105 electronic nose sample data that were acquired from 7 infestation durations for the insect prevalence prediction. We chose 4 rough rice samples randomly from each treatment of different infestation durations as the training set, and the remaining one sample from each treatment of different infestation durations as the test set. Thus, the training set of each treatment had 28 samples (4 samples per treatment × 7 infestation durations = 28), and the test set of each treatment had 7 samples (1 sample per treatment × 7 infestation durations = 28). Thus, this experiment had 84 samples (28 samples per infestation duration × 3 treatments = 84) in the training set, and 21 samples (7 samples per infestation duration × 3 treatments = 21) in the test set, in total. After repeated training, the BPNN model parameters of LD, MD, and HD were as follows: the nerve cell numbers of the hidden layer was 21, 18, and 21, respectively; all of the treatments utilized the BPNN “trainlm” training algorithm; all of the treatments had BPNN hidden layer numbers of 2; all of the BPNN output layers used the “tansig” function; and the BPNN iteration numbers and sampling frequencies were 2000 and 25, respectively, for all treatments.

**Figure 2:**
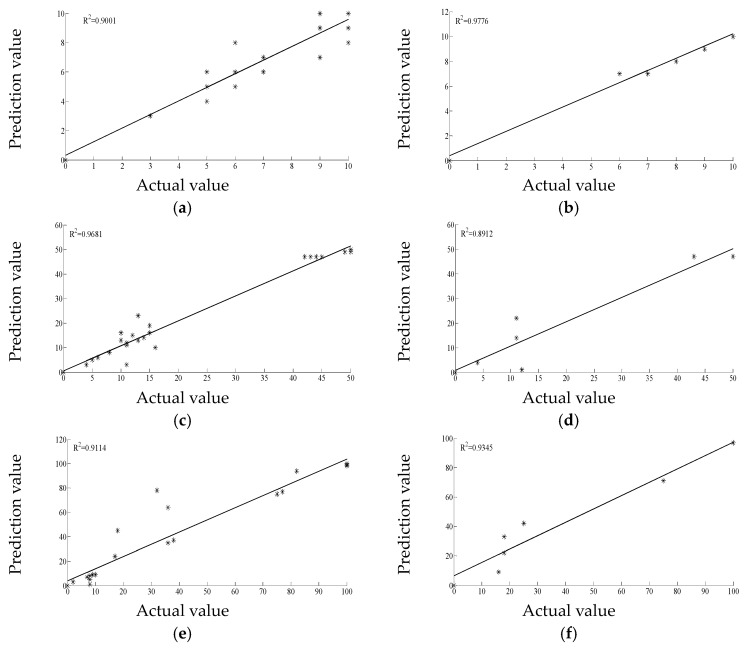
Matched curves of the BPNN prediction and actual values for infested rough rice insect amounts; (**a**) matched curves for the LD training set; (**b**) matched curves for the LD test set; (**c**) matched curves for the MD training set; (**d**) matched curves for the MD test set; (**e**) matched curves for the HD training set; (**f**) matched curves for the HD test set.

The matched curves of the BPNN prediction values and actual values for the infested rough rice live insect amounts are shown in Figure 2. The determination coefficient (R^2^) of the matched curves of the BPNN prediction and actual values for training sets of LD, MD, and HD are 0.9001, 0.9681, and 0.9114, respectively. The determination coefficient (R^2^) of the matched curves of the BPNN prediction and actual values for test sets of LD, MD, and HD are 0.9776, 0.8912, and 0.9345, respectively. All of the BPNN insect prevalence predictions had good imitative effects (all of the matched curves had R^2^ values greater than 0.89). These results indicate that electronic noses can effectively predict insect amounts in stored grain.

The BPNN prediction results for the LD, MD, and HD test sets are shown in Table 4. The average difference values and average relative errors of the prediction and actual values for LD are the smallest, are intermediate for MD, and are highest for HD. The correct prediction numbers of LD, MD, and HD were 4, 2 and 1, respectively. Thus, the prediction power for LD is the best, is intermediate for MD, and is worst for HD. According to the experimental results, we can infer that the electronic nose method is most suitable for the insect prevalence prediction in stored grain during the insect infestation first occurs.

## 4. Discussion

This paper explored the feasibility of using an electronic nose for inferring infestation duration and prevalence in stored grains. The results show that electronic noses can effectively predict the stored grain infestation duration and insect prevalence.

During injury, live insect numbers of all three infestation categories decreased gradually. Furthermore, the higher the degree of infestation, the more rapid the decrease in live insect number. Previous research results of Zhou et al. indicated that [25] serious internecine phenomena will occur if the stocking density is too high, which will cause a gradual decrease in the insect survival rate. However, if the insect survival rate reaches a particular value, then the survival rate will tend to stabilize. The results of Zhou et al. are consistent with the changes in insect number found in this experiment. 

LDA, FCM, and K-means results show that the recognition effects of MD and HD rough rice were better than recognition effects of LD rough rice, which indicates that the heavier the insect infection is, the more special the odors [11] in the storage environment will be. In addition, FCM performed better than K-means for the infestation duration prediction. This is because [26] K-means distribute each sample to only one class and cannot distribute samples to several classes at the same time. This analysis method is sensitive to noise and abnormal values and can easily be caught in local maxima during optimization. However, when using FCM for analysis, each sample can be distributed to several classes at the same time [27]. Compared with K-means, FCM can avoid certain problems, such as local optimization maxima.

The results of BPNN show that predicting insect prevalence in stored rough rice based on electronic nose data is feasible for all insect infectious degrees. The predictive abilities of LD were the best, were intermediate for MD, and were the worst for HD. This may be because interference during the detection period increases with additional insects in a fixed size space, where interference may arise from the smell of dead insects and the metabolism of live insects. Research into how to overcome this interference during insect prevalence detection in stored grain is a valuable future direction to consider for new research. In practical insect prevalence detection, the infected situation of an entire granary can be repeatedly evaluated by the results of a random sample, with the detection method of an electronic nose combined with a BPNN model.

## 5. Conclusions

This experiment used an electronic nose (PEN3) to sample stored rough rice at different red flour beetle infestation degrees at different time points (2 days before infestation, 2 days, 7 days, 13 days, 22 days, 28 days, and 36 days after infestation). The manual work detection method was used to count the live insect numbers at the same time. The experimental results are as follows.

(1) The results of the manual work detection method show that live red flour beetle numbers in all of the LD, MD, and HD treatments gradually decreased. We can infer that the insect number natural declines slowly when the insect population in storage grain is under 13 individuals/60 g, which is the necessary period for manual insect control.
(2) LDA performed well for predicting the infestation duration in MD and HD, but poorly for LD. K-means correctly predicted the infestation duration of LD, MD, and HD 88.57%, 91.42%, and 91.42% of the time, respectively. FCM correctly predicted the infestation duration of LD, MD, and HD 94.29%, 100%, and 100% of the time, respectively.
(3) Matched curves of the BPNN prediction and actual values for the infested rough rice insect amounts of the LD, MD, and HD samples indicated that electronic noses can effectively predict the amount of insects in stored grain (all of the matched curves had R2 values greater than 0.8). We can also infer that the electronic nose method is the most suitable method for the insect prevalence prediction of stored grain once insects first infest grains.
(4) This experiment supports the feasibility of predicting the infestation duration and insect prevalence in stored grains based on electronic nose measurements and provides a reference for the intelligent monitoring of stored grain insect infestations.

## Figures and Tables

**Table 1:** Insect number change in different infestation degrees in rough rice at different times.

	−2 d	2 d	7 d	13 d	22 d	28 d	36 d
**NT**	0	0	0	0	0	0	0
**LD**	0	10	9	8	7	6	6
**MD**	0	50	43	13	12	11	10
**HD**	0	100	77	33	13	11	11

**Table 2:** K-means for duration of different infested degree rough rice.

	Correct Classified Sample Number							Accuracy/%
	−2 d	2 d	7 d	13 d	22d	28d	36 d	
**LD**	4	5	5	5	3	4	5	88.57
**MD**	5	5	4	5	3	5	5	91.42
**HD**	5	5	5	3	4	5	5	91.42

**Table 3:** Fuzzy c-means analysis (FCM) for duration of different infestation degrees of rough rice.

	Weighted Value m	Correct Classified Sample Number							Accuracy/%
		−2 d	2 d	7 d	13 d	22 d	28 d	36 d	
**LD**	1.6	4	5	5	5	4	5	5	94.29
**MD**	2	5	5	5	5	5	5	5	100
**HD**	1.5	5	5	5	5	5	5	5	100

**Table 4:** Back-propagation artificial neural network (BPNN) insect prediction results for the test sets of LD, MD, and HD.

Sample Number	LD			MD			HD		
	Prediction Value/Actual Value	Difference Value	Relative Error %	Prediction Value/Actual Value	Difference Value	Relative Error %	Prediction Value/Actual Value	Difference Value	Relative Error %
1	0/0	0	0	0/0	0	0	0/0	0	0
2	10/10	0	0	47/50	3	6	97/100	3	3
3	9/9	0	0	47/43	5	11.6	71/75	4	5.33
4	8/8	0	0	4/4	0	0	42/25	17	68
5	7/6	1	14.28	14/11	3	27.27	18/22	4	18.18
6	7/7	1	0	1/12	11	91.7	9/16	7	43.75
7	6/7	1	14.28	22/11	11	1	33/18	15	8.33
Average		0.43	4.08		4.71	19.55		7.14	20.94
